# Structural Characteristics and Multiple Bioactivities of *Volvariella volvacea* Polysaccharide Extracts: The Role of Extractive Solvents

**DOI:** 10.3390/foods12234357

**Published:** 2023-12-02

**Authors:** Jun Wang, Changyu Zhao, Ping Li, Lei Wang, Songnan Li

**Affiliations:** 1 School of Tourism and Cuisine, Yangzhou University, Yangzhou 225127, China; 007232@yzu.edu.cn (J.W.);; 2 Key Laboratory of Functional Foods, Ministry of Agriculture and Rural Affairs, Guangdong Key Laboratory of Agricultural Products Processing, Sericultural & Agri-Food Research Institute, Guangdong Academy of Agricultural Sciences, Guangzhou 510610, China; 3 College of Food Science and Engineering, Henan University of Technology, Zhengzhou 450001, China; 4 Joint International Research Laboratory of Agriculture, Agri-Product Safety of the Ministry of Education of China, Institutes of Agricultural Science and Technology Development, Yangzhou University, Yangzhou 225009, China

**Keywords:** *Volvariella volvacea*, extractive solvent, rheological property, functional property, hypoglycemic activity

## Abstract

The chemical structures and functional properties of plant-based polysaccharides are critically influenced by extractive solvents, but their roles are not clear. In this study, the structural characteristics and multiple bioactivities of *Volvariella volvacea* polysaccharides (VVPs) subjected to water (VVP-W), alkalis (sodium hydroxide, VVP-A), and acids (citric acid, VVP-C) as extractive solvents are investigated systematically. Of the above three polysaccharides, VVP-W exhibited the highest molecular weights, apparent viscosity, and viscoelastic properties. Functional analyses revealed that VVP-C had an excellent water-holding capacity, foaming properties, and emulsifying capacity, while VVP-A exhibited a promising oil-holding capacity. Moreover, VVP-C displayed strong inhibitory effects on α-amylase and α-glucosidase, which could be attributed to its content of total phenolics, proteins, and molecular weights. These findings have important implications for selecting the appropriate extraction techniques to obtain functional polysaccharides with targeted bioactive properties as food additives.

## 1. Introduction

People have consumed mycelia as a food source and nutritional supplement for millennia because of its delicate flavor, aroma, and healing properties. They are essential for meeting our daily nutritional needs since they contain high-quality proteins, unsaturated fats, dietary fibers, minerals, and essential vitamins [1]. Mushrooms have a high content of functional homopolysaccharides, such as β-glucans and/or α-glucans, exhibiting a promising biological response [2]. Among these, the tropical/subtropical edible mushroom species *Volvariella volvacea* (VV) is predicted to be produced in annual quantities of 330,000 tons in China alone, which accounts for over 80% of the global production [3,4]. On a dry basis, VV has a protein content of roughly 25.9%–29.6% and a fat content of 2.24%–3.60% [3,5]. Furthermore, VV has a high vitamin C content of 200 mg per 100 g in its fresh state [6]. In addition to its high nutritional value, VV has been found to contain a range of bioactive compounds that exhibit positive health effects. These include immune-regulating properties, scavenging capabilities against reactive oxygen species, and anti-aging functions [7].

In recent years, researchers have developed various methods for the extraction of fungal polysaccharides. Among them, the hot water extraction method has been widely applied due to its simplicity and safety and being environmentally friendly. However, this extraction process requires prolonged high-temperature heating, which not only leads to an excessive energy consumption and polysaccharide degradation, but also may generate unnecessary side reactions and darken the color of the extracted polysaccharides with the increase in the temperature. To address this issue, researchers have employed different extraction methods using sustainable unconventional processes for fungal polysaccharides, including acidic, alkaline, and salt solutions, along with various auxiliary techniques, such as ultrasound-assisted and microwave-assisted extractions. It has been possible to extract and isolate bioactive polysaccharides effectively from the fruit bodies of both wild and cultivated VV. The bioactivity of these VV polysaccharides (VVPs) is attributed to their distinct molecular weights and chemical structures, and the extraction technique is often crucial to their identification and application [8]. Selecting the right extraction method is crucial, as it relies on the understanding of the fungal cell wall structure, specifically the role of VVPs as key structural components [9]. Water extraction is a well-established technique frequently used in laboratory and industrial processes to extract polysaccharides. Notable benefits of this approach are its ease of processing and affordability and being environmentally friendly [10]. For polysaccharide extraction, additional solvents (such as alkalis and acids) are frequently used in addition to water. Fungal cell walls may be efficiently broken down by these solutions, with the outer layer breaking down first and then the inner layer [11]. Liu et al. (2014) isolated polysaccharides from *Jinqian* mushrooms using alkali extraction (1.25 M NaOH and 0.05% NaBH_4_); they observed unique antioxidant properties and a high yield of up to 9.78% [12]. Using a 1% HCl solution, the polysaccharides isolated from *Cordia dichotoma* G showed good flow characteristics and 86.24% (*w*/*w*) of the total polysaccharide content [13]. When Yao et al. (2017) compared the method used to extract polysaccharides from *Laminaria japonica*, they discovered that using citric acid during the extraction process resulted in the highest extract yield (~11% dry weight) and bile salt adsorption rate (~59% dry weight), as well as improved antioxidant and free radical scavenging capabilities [14].

The role of extractive solvents in the structural features and functional properties of VVPs remain unclear. However, multiple attempts have shown that these solvents significantly impact the extract yield, physicochemical properties, structural characteristics, biological activities, and functionalities of polysaccharides. This study involves a systematic investigation of the structural characteristics (chemical composition, surface microstructure, and functional group structure) and multiple bioactivities (rheological properties, water- and oil-holding capacities, foaming properties, emulsion properties, and the inhibition of α-Amylase and α-glucosidase) of VVPs subjected to extractive solvents that included water (VVP-W), alkalis (sodium hydroxide, VVP-A), and acids (citric acid, VVP-C).

## 2. Materials and Methods

### 2.1. Materials and Chemicals

VV was purchased from a local supermarket (Yangzhou, China). We obtained dextran standards, bovine serum albumin, p-nitrophenyl-α-d-glucopyranoside, α-amylase, and α-glucosidase from Sigma-Aldrich (St. Louis, MO, USA). Aladdin (Shanghai, China) provided the monosaccharide standards. All of the chemicals and reagents were analytical grade.

### 2.2. Extraction of Polysaccharides

Fresh VV specimens underwent freeze-drying until a consistent weight was achieved. Then, using an electric grinder, they were pulverized into a powder (approximately 0.15 mm size) to extract the polysaccharides. We weighed the item in powder form and mixed it with anhydrous ethanol. We conducted two rounds of defatting by removing the fat at 55 °C for 3 h. We filtered the mixture and dried it at 60 °C for further use.

Three different extraction methods of polysaccharides were previously reported, albeit with some modifications [15]. In detail, 100 g samples for water extraction (VVP-W) were extracted with 2 L deionized water at 95 °C for 3 h; 100 g samples for alkali extraction (VVP-A) were extracted with a 2 L NaOH solution (pH = 9) for 3 h; and 100 g samples for acid extraction (VVP-C) were extracted with a 2 L citric acid solution (pH = 2) at 95 °C for 3 h. Each extract was concentrated and deproteinized using Sevage reagent (N-butanol/chloroform, *v*/*v* = 1:4), and the remaining reagent was removed under low pressure. The VVPs underwent precipitation with three times the volume of absolute ethanol (4 °C for 24 h) prior to being dialyzed (molecular mass cut off: 1000 Da) against deionized water for 72 h and then freeze-dried.

### 2.3. Chemical Composition of Polysaccharides

The phenol–sulfuric acid method and the Bradford method based on Coomassie blue were used to evaluate the total polysaccharides and total protein content from VVP-W, VVP-A, and VVP-C [15,16]. Their total phenolic contents without extraction were determined by the Folin–Ciocalteu antioxidant capacity assay in a NaOH-added isobutanol–water medium with gallic acid as the reference [9].

An ICS 3000 ion chromatography system (Thermo Fisher Scientific; Waltham, MA, USA) was used to analyze each of the individual fractions obtained from the extracts, and the monosaccharide compositions of the extracts were determined using the method described by Wang et al. [17]. The molecular weights of the samples were determined using the HPLC-RI system (Shimadzu, LC-10A; Osaka, Japan) with a BRT105-104-102 (8 × 300 mm) column (BoRui Saccharide Biotech Co., Ltd., Yangzhou, China). Chromatographic conditions: column temperature of 40 °C, mobile phase of 0.05 M NaCl, and flow rate of 0.6 mL/ min. As the system precorrection, the logarithm of the mean molecular weight of PEG standard was used to plot the curve; the data were analyzed to obtain the weight-average molecular weight (M_w_) [18].

### 2.4. Scanning Electron Microscopy (SEM)

A Quanta 250 FEG SEM (FEI Technologies Inc., Hillsboro, OR, USA) was used to examine the surface microstructure of the various polysaccharides. The samples were placed on aluminum sheets, immobilized with double-sided tape, then coated with gold, and observed at 10 kV voltage to accelerate the electrons [19].

### 2.5. Fourier-Transform Infrared (FTIR) Spectroscopy

The KBr pellet method was used to prepare the polysaccharides, and the IARffinity-1S FTIR spectrometer (Thermo Fisher Scientific; Waltham, MA, USA) was used to record the spectra, ranging from 400 cm^−1^ to 4000 cm^−1^ [20].

### 2.6. Rheological Measurements

An HR-1 Discovery Hybrid Rheometer from TA Instruments (New Castle, DE, USA) was used to evaluate the rheological properties. Sample solutions containing 8 mg/mL of deionized water were placed between 40 mm diameter steel plates, spaced 1.0 mm apart, to measure the apparent viscosity. The shear rates at which the measurements were conducted ranged from 1 to 100/s. The Power–Law model was used to fit the flow curves [21]:

Power–Law model: η = k(γ)^n^ (1)

where η is the apparent viscosity, k is the viscosity coefficient (Pa·s^n^), γ is the shear rate (s^−1^), and n is the flow behavior index.

A rheometer was used to perform dynamic oscillatory experiments using a parallel plate (40 mm diameter and 1.0 mm gap), according to a previous study [22]. The storage modulus (G′) and loss modulus (G″) of the polysaccharides were determined by frequency sweeps conducted at 25 °C with oscillation frequencies ranging from 1 to 100 rad/s. An oscillation strain sweep was performed at a constant frequency of 1 Hz to find the linear viscoelastic region.

With a heating/cooling rate of 2 °C /min, we conducted temperature ramp experiments from 20 °C to 80 °C and temperature ramp-down experiments from 80 °C to 20 °C. The frequency of oscillation was fixed at 1 Hz. A 1% strain and parallel plates (40 mm diameter) were used for the measurements [23].

### 2.7. Functional Properties

#### 2.7.1. Water- and Oil-Holding Capacities

The method described by Jeddou et al. (2016) was partially modified to test the water-holding capacity (WHC) and oil-holding capacity (OHC) [24].

In a centrifuge tube, 150 mg of the material was mixed with 15 mL of distilled water (1%, *w*/*v*) for the WHC. The tube was weighed to determine the initial weight. After that, the mixture was swirled gently for 5 min with a magnetic stirrer and left to settle for 30 min without any disturbance. After that, the tube was centrifuged at 3000 rpm for 25 min to separate the solid material from the liquid portion (supernatant). After carefully removing the supernatant, the solid-sample-containing tube was placed uprightly to drain the excess water at 50 °C for 25 min. The experiment was conducted in triplicate to ensure accuracy and produce reliable results. The WHC was calculated as follows:

(2) $$\mathrm{WHC}\;(g/g)=\frac{\mathrm{Water}\;\mathrm{absorbed}\;\mathrm{weight}}{\mathrm{Sample}\;\mathrm{weight}}$$

A magnetic stirrer mixed 0.2 g of samples and 10 mL of soybean oil for 1 min to determine the OHC. After 30 min of stirring, the mixture was centrifuged at 3000 rpm for 25 min to remove the supernatant. Filter paper was used to invert the centrifuge tube and drain the excess oil for 30 min. The experiment was conducted in triplicate. The OHC was calculated as follows:

(3) $$\mathrm{OHC}\;(g/g)=\frac{\mathrm{Oil}\;\mathrm{absorbed}\;\mathrm{weight}\;}{\mathrm{Sample}\;\mathrm{weight}}$$

#### 2.7.2. Foaming Properties

The method described by Rezaei et al. (2016) was used to measure the foaming capacity (FC) and foaming stability (FS) [25]. High-speed shear homogenization at 10,000 rpm for 1 min at room temperature was applied to polysaccharide solutions at different concentrations (0.25%, 0.5%, 1.0%, and 2.0% *w*/*v*). FS was assessed after 30 min, and FC was calculated using the foaming volume measurements at t = 0 s. FS is expressed as the percentage of foam volume after 30 min, whereas FC is the percentage of volume increase after 0 min of homogenization. The calculations for FC and FS are as follows:

(4) $$\mathrm{FC}\;(g/g)=\frac{\mathrm{Initial}\;\mathrm{foam}\;\mathrm{volume}}{\mathrm{Total}\;\mathrm{suspension}\;\mathrm{volume}\;}\times 100$$

(5) $$\mathrm{FS}\;(g/g)=\frac{\mathrm{Final}\;\mathrm{foam}\;\mathrm{volume}}{\mathrm{Total}\;\mathrm{suspension}\;\mathrm{volume}}\times 100$$

#### 2.7.3. Emulsion Properties

The modified method used to measure the emulsion capacity (EC) and emulsion stability (ES) of the VVPs was based on the study conducted by Jindal et al. (2013) [23]. VVP solutions at different concentrations (0.25%, 0.5%, 1.0%, and 2.0% *w*/*v*) were mixed with 3 mL of soybean oil (Yihai Kerry Group, Singapore) at room temperature. A high-speed shear homogenizer (T25, IKA Co., Staufen im Breisgau, Germany) was used to homogenize the mixture for 1 min at 1000 rpm for 10 min to separate the phases. The EC was calculated using the following formula:

(6) $$\mathrm{EC}\;(\%)=\frac{\mathrm{Emulsion}\;\mathrm{volume}\;}{\mathrm{Total}\;\mathrm{volume}\;}\times 100$$

The emulsion was subjected to a heat stability test to determine the ES. After heating at 80 °C for 30 min, the emulsion was allowed to cool to room temperature. The phases were separated by centrifuging the emulsion at 1000 rpm for 10 min. The ES was calculated using the following formula:

(7) $$\mathrm{ES}\;(\%)=\frac{\mathrm{Final}\;\mathrm{emulsion}\;\mathrm{volume}}{\mathrm{Total}\;\mathrm{volume}\;}\;\times \;100$$

### 2.8. α-Amylase and α-Glucosidase Inhibition Assays

The measurement of the α-amylase inhibition was conducted using the method described by Meng et al. (2016) [26]. The determination of the inhibitory activity of α-glucosidase was determined using the method described by Wang et al. (2018) [27].

### 2.9. Statistical Analysis

The experiments were conducted in triplicate, and the results are presented as the mean ± standard deviation. A one-way analysis of variance and Duncan’s test were used in the statistical analysis, which was conducted using SPSS software (version 19.0, SPSS Inc., Chicago, IL, USA) to evaluate the differences and determine the significance of the comparisons (*p* < 0.05).

## 3. Results

### 3.1. Chemical Compositions of VVPs

The extraction yields and physicochemical properties of the three VVPs that were extracted using different techniques from the fruit body of VV are listed in Table 1. The extraction yields and physicochemical properties showed significant differences according to the extraction solvents. Depending on the extraction solvent utilized, the yield of VVPs varied and ranged from about 5.15% to 7.68%. Among these VVPs, the yields from VVP-A (7.68% *w*/*w*) and VVP-C (6.81% *w*/*w*) were higher than that from VVP-W (5.15% *w*/*w*), consistent with the results of a previous study [28]. Alkaline solutions work better during extraction to dissolve the hydrogen bonds that bind cellulose and hemicellulose in the cell walls. As a result, insoluble polysaccharides are more easily released from the cell walls and transformed into soluble polysaccharides [29]. The alkaline solution thus showed the highest yield for polysaccharide extraction from VV among the three extraction solvents. VVP-C had a lower yield than VVP-A at the same extraction temperature and duration. This may be explained by the fact that polysaccharides are vulnerable to acid hydrolysis during the extraction process in acidic media, mainly when the extraction temperature is 95 °C. Consequently, short-chain oligosaccharides or monosaccharides are produced by hydrolyzing the extracted long-chain polysaccharides [9]. A reduced yield of VVP-C results from the decreased propensity to precipitate during the following ethanol precipitation process. Polysaccharides are released when the hydrogen bonds separating cellulose and hemicellulose in plant cell walls are broken in an alkaline environment. Citric acid can break down pectin components in cell walls, making polysaccharides easier to dissolve. Conversely, water produced the lowest extraction of polysaccharides because it can only dissolve long-chain soluble polysaccharides. This is in line with the results obtained by Yan et al. (2018), who extracted polysaccharides from *Hericium erinaceus* using several extraction solvents [9].

Table 1 further shows the total sugar contents of VVP-W, VVP-A, and VVP-C ranging from 81.65% to 87.69%. This result indicates the high purity of polysaccharides, which should be attributed to the specialized extraction process with deproteinization, ethanol-defatting, and effective dialysis [9]. In addition, their protein contents were relatively low (1.41%–2.77%), because the three mushroom polysaccharides are deproteinized during solvent extraction, which significantly lowers the amount of free proteins present. However, compared to VVP-W, the protein contents of VVP-A and VVP-C were noticeably lower. This is because proteins and polysaccharides have more easily broken hydrogen bonds in acidic or alkaline solutions, which results in a higher protein extraction during the alcohol precipitation process. Since uronic acids are absent from three polysaccharides, they may be considered neutral polysaccharides. 

Additionally, their polyphenolic content was also determined due to the potential role of polyphenols in physicochemical properties (such as emulsification and rheological properties) and bioactivity, and a small amount of polyphenols, bound to polysaccharides, is hard to eliminate completely [10]. The three VV polysaccharides extracted using various solvents presented micro-levels of polyphenolic compounds (18.41–26.04 mg GAE/g). The outcomes from the previously described research suggest that the particular extraction technique and solvent selection may have contributed to the observed variations in the polysaccharides. These findings are consistent with the results of previous studies conducted by Wang et al. (2014) [30] and Kou et al. (2022) [31].

### 3.2. Molecular Structure of VVPs

**Figure 1:**
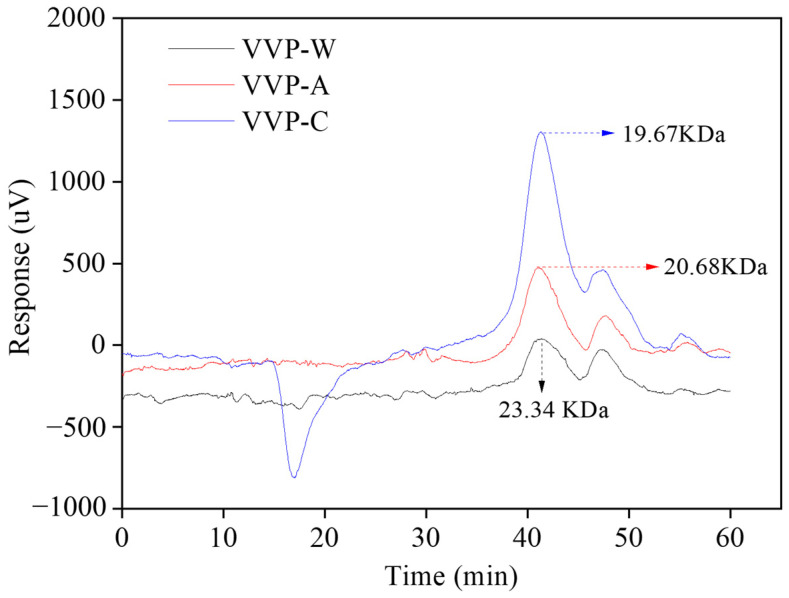
Molecular weight distribution of the VVPs.

The molecular weights and constituent monosaccharides of natural polysaccharides play an important role in their functionalities and bioactivities [32]. As a result, the molecular weights of the VVPs extracted using various techniques were ascertained. The molecular weight measurements were conducted using a standard curve equation derived from dextran standards. LogM_w_ = −0.1985V + 12.509, where M_w_ is the molecular weight and V is the elution volume. A good association between the elution volumes and the molecular weights of the VVPs was indicated by the equation’s high correlation coefficient of 0.9964. The HPLC-RI chromatograms produced by analyzing the VVP extracts are displayed in Figure 1. Each extract yielded a single elution signal, indicating that the VVPs were homogenous polysaccharides. VVP-W, VVP-A, and VVP-C had molecular weights of 23.34 × 10^3^ Da, 20.68 × 10^3^ Da, and 19.67 × 10^3^ Da, respectively. The results of our study on the molecular weights of polysaccharides are different from those published by Cui et al. (2018) [33]. Variations in the extraction conditions, including time and temperature, could have caused this discrepancy. On the other hand, the molecular weights of the VVPs extracted using acid and alkali were lower than those obtained with hot water, suggesting that the polysaccharide chains were broken down and destroyed during the acid and alkaline extraction processes. Some high M_w_ fractions may be converted into lower M_w_ fractions during this process, increasing the number of low M_w_ fractions in the VVP molecules. Thus, the polysaccharide chains appear to have broken down and changed as a result of the acid and alkaline extractions, which has an impact on the molecular weight distribution in the VVPs. The same results were also obtained in previous studies [30]. For instance, using hot water extraction, two conspicuous peaks with average molecular weight values of 320,000 kDa and 49.2 kDa were seen in polysaccharides extracted from *Phellinus linteus*. Based on peak area analysis, the significant fraction was composed of around 63% of the total polysaccharides. It was distinguished from the other fractions by having a higher molecular weight. On the other hand, the polysaccharides from *Phellinus linteus* showed two different groups with M_w_ values of 975,000 kDa and 13.9 kDa, which were produced by acid extraction. However, most of the composition comprised roughly 60% of the lower-molecular-weight component.

### 3.3. Monosaccharide Compositions of VVPs

**Figure 2:**
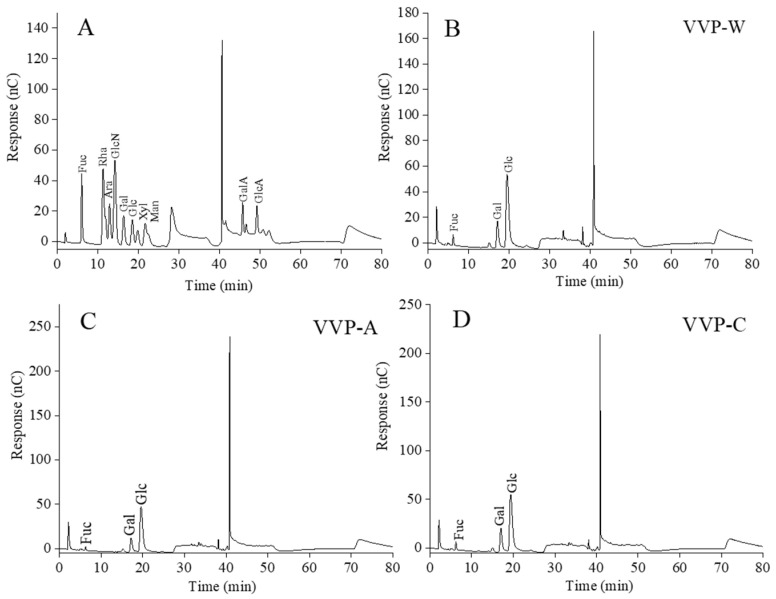
The ion chromatography profiles of a mixture of the monosaccharide standards (**A**) and VVPs (**B**–**D**).

Trifluoroacetic acid was used to hydrolyze VVPs, fully converting their constituents into monosaccharides. The results of the ion chromatography analysis of these monosaccharides are shown in Figure 2 and Table 1. After comparing the three VVPs with monosaccharide standards, it was discovered that the molar percentages of fucose, glucose, and galactose varied. This finding suggests that the VVPs extracted from *Volvariella volvacea* are heteropolysaccharides with unique chemical compositions. Interestingly, the most common monosaccharide in these VVPs was glucose. D-glucose, D-mannose, and D-galactose are present in the endopolysaccharides extracted from *Volvariella volvacea* using hot water, according to the report by Cui et al. (2018) [29]. Among these, D-glucose is the most prevalent monosaccharide, which is significant. Cui et al. (2020) also found significant disparities between the results of our study and the monosaccharide compositions of a macromolecular α-glucan extracted with hot water from the fruits of *Volvariella volvacea* [3]. As a result, variations in the raw material source, extraction techniques, and isolation protocols may be to blame for the diversity in the monosaccharide compositions of VVPs.

### 3.4. Surface Morphologies of VVPs

**Figure 3:**
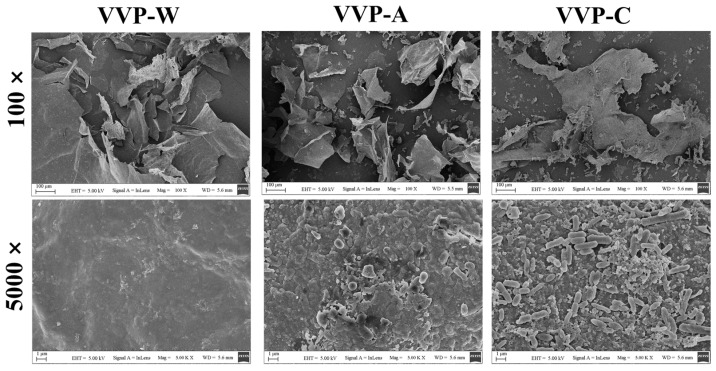
SEM images of VVPs.

The surface morphologies of three different VVPs (VVP-W, VVP-A, and VVP-C) are shown as SEM images in Figure 3 at magnifications of 100× and 5000×. The SEM images clearly show the considerable variations in shape and size among these VVPs, which were extracted using various solvents. After being removed with hot water, VVP-W had a vast, rough surface with a considerable quantity of aggregates that formed vast tracts of polysaccharides. The surfaces of VVP-A and VVP-C were comparatively loose and flaky, suggesting that the polysaccharides may have been partially disrupted and reduced in size due to the use of citric acid as the extraction solvent. This discovery is aligned with the outcomes obtained from the HPLC analysis. The alkali solution used to extract VVP-A affected the cell walls of the fruiting bodies more severely. This resulted in cell disintegration and increased the area where the solid and liquid phases come into contact. Consequently, Figure 3 shows that the surface of VVP-A had a reticular layer, nonuniform size and a somewhat rough texture.

### 3.5. Organic Groups of VVPs

**Figure 4:**
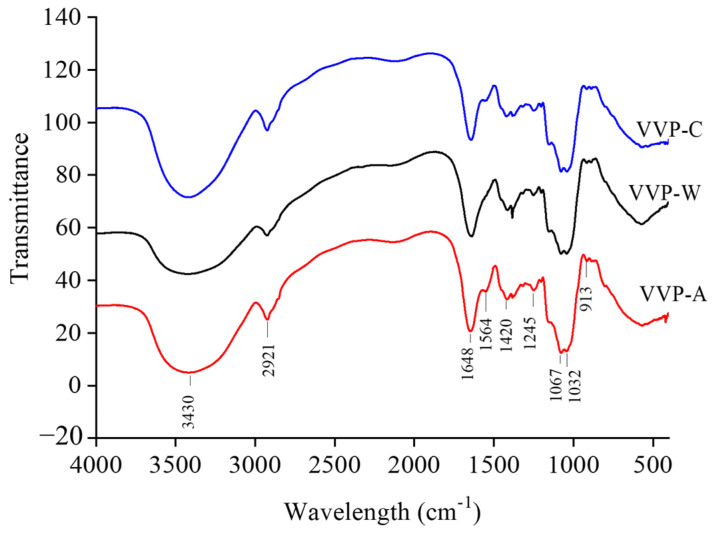
FT-IR spectra of VVPs.

The standard method for identifying distinct organic groups in polysaccharides is FT-IR spectroscopy. The FT-IR spectra of VVP-W, VVP-A, and VVP-C are shown in Figure 4. The research revealed no appreciable variations in the distinctive organic groups of VVPs produced from varied extraction solvents. The stretching vibration of the O-H bonds, commonly seen in hydroxyl groups, was identified as the cause for the broad and robust absorption peak seen in the FT-IR spectra at approximately 3330 cm^−1^ [34]. However, the weak band at about 2921 cm^−1^ was linked to the stretching vibration of C-H bonds, suggesting that the samples included carbon–hydrogen groups [35]. The FT-IR spectrum of VVPs does not show an absorption peak near 1730 cm^−1^, indicating the absence of uronic acid [36]. The absorbance shows the presence of bound water at 1650 cm^−1^ [37]. This implies that the sample and water molecules interact. The properties of the CH_2_ deformation mode are responsible for the absorption peak at 1414 cm^−1^ [37]. The band in the spectrum located at 1243.14 cm^−1^ is probably caused by the C-OH group vibration [38,39]. The polysaccharides’ pyranose form is indicated by the peaks at 1081 cm^−1^ and 1023 cm^−1^ [38]. This suggests that the sugar units in the VVPs are organized in a pyranose ring structure, which is a typical polysaccharide configuration.

### 3.6. Rheological Properties of VVPs

#### 3.6.1. Apparent Viscosity

**Figure 5:**
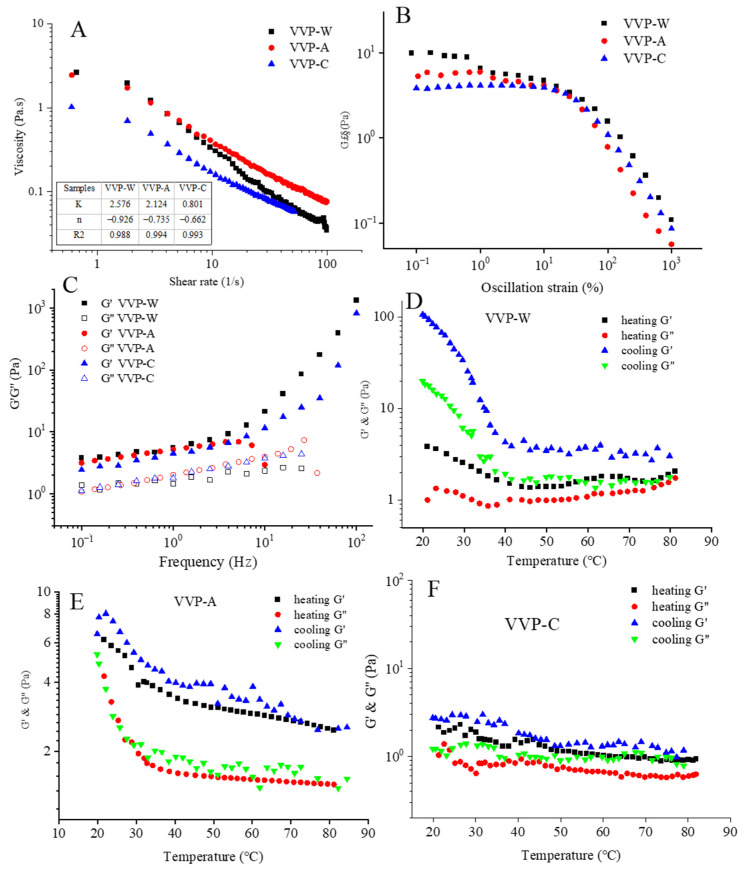
Apparent viscosity at a shear rate range from 0.1 s^−1^ to 100 s^−1^ at 25 °C (**A**); linear viscoelastic region (**B**); storage (G′) and loss (G″) moduli at the concentration of 1% (**C**); and variation in G′ and G″ during heating and cooling by 2 °C/min (**D**–**F**) of VVPs.

The steady-state flow curves of VVPs at varied shear rates are shown in Figure 5A. The apparent viscosity of the polysaccharides steadily decreased with the increase in the shear rate, suggesting that all VVP solutions show shear thinning and pseudoplastic fluid behavior. According to a previous report, the reduction in polymer chain entanglement and the rise in the shear rate are the leading causes of polysaccharide shear thinning behavior [40]. The polysaccharide molecules can quickly realign along the flow direction, leading to a notable reduction in the apparent viscosity. The steady flow curves were fitted with the Power–Law model for analysis, and Figure 5A summarizes the corresponding parameters. The VVP rheological behavior was well described by the Power–Law model, as evidenced by the R^2^ values of VVPs being greater than 0.9. The k-value represents the viscosity consistency. Viscosity trends can be seen using this relationship, which shows that greater k-values correspond to an increase in the viscosity. The *k*-values in this study for VVP-W, VVP-A, and VVP-C were 2.576, 2.124, and 0.801 Pa·s, respectively, showing that the viscosity of polysaccharides increases with the molecular weight. Higher viscosity polysaccharides have been shown to have improved bile-salt- and glucose-binding capabilities, which is advantageous for lowering blood sugar and fat levels [22]. Therefore, metabolic syndrome may be treated with high-molecular-weight VV polysaccharides.

The n-value is also related to the degree of pseudoplasticity. Lower n-values are associated with higher pseudoplasticity fluids. The fluid is an ideal (Newtonian) fluid when n = 1. The fluid is non-Newtonian and exhibits shear thinning behavior when n is more significant or less than 1. Significantly below 1, the n-values of the different VVPs showed a strong pseudoplasticity, especially for lower-molecular-weight polysaccharides, like VVP-A (−0.735) and VVP-C (−0.662). These findings suggest thickening agents with a higher molecular weight can be used in the functional food business. This includes the possibility of using them to thicken diabetic-specific beverages. Furthermore, the pseudoplastic qualities of liquid foods make pumping more accessible and add to their pleasant mouthfeel [21]. Consequently, the food industry may employ VVP-W and VVP-A as thickening agents.

#### 3.6.2. Linear Viscoelastic Region

Identifying the linear viscoelastic zone is crucial since it keeps the samples from being readily destroyed. In this study, stress levels ranging from 0.1% to 1000% were applied to various concentrations of VVP solutions (ranging from 0.5% to 2.0%). Figure 5B shows the outcomes of these measurements. Following an analysis of the data, dynamic oscillatory measurements on each sample were decided upon using a 1% strain change. This strain level was chosen because it lies within the linear viscoelastic region, where the material reaction may be considered linear and reversible. This guarantees that the samples will not experience undue deformation or stress, which could result in structural damage.

#### 3.6.3. Oscillatory Properties

The storage modulus G′ and the loss modulus G″ are commonly used to assess the viscoelastic properties of polysaccharide solutions and gels, respectively. A solution is primarily viscous and behaves more “liquid-like” when G′ is lower than G″. On the other hand, the system exhibits common elastic properties if G′ is more significant than G″. Figure 5B shows the mechanical spectra of the VVPs in the linear viscoelastic zone. Additionally, the viscoelastic behavior of the VVP solutions at the same concentrations at a strain of 1% is shown in Figure 5C. Based on an analysis of the linear viscoelastic region seen in Figure 5B, this strain value was chosen. As Figure 5C illustrates, a gel-like behavior was shown by both VVP-W and VVP-C, with G′ being consistently higher than G″. In the case of VVP-A, at low frequencies (0.1–10 Hz), the storage modulus (G′) was greater than the loss modulus (G″). G″ predominated at high frequencies (10–100 Hz), resulting from the disruption of the structured nature of the polymer. The frequency at which the G′ and G″ of VVP-A intersected was 10 Hz. This implies that, when the frequency increased, the VVP-A solution behavior changed from being elastic to more viscous. The above behaviors are aligned with the entanglement properties commonly reported in uncharged random coil polysaccharides [41]. Numerous other polysaccharide solutions, such as galactomannans, guar, and locusta, have also shown similar behaviors [42].

The thermal stability of the rheological properties of VVPs was assessed with the help of cooling and heating measures. The G′ of VVP-W, VVP-A, and VVP-C was more significant than their G″ in the 20–80 °C temperature range, as illustrated in Figure 5D–F. G″ also drastically dropped with the increase in the temperature while displaying a gel-like behavior. Both the G′ and G″ of VVP-W and VVP-A significantly increased during the cooling process compared to the heating curves, indicating the construction of a more extensive network of cross-linking structures. Additionally, an accelerated increase in G′ and G″ values at about 40 °C was seen in the cooling curves of VVP-W and VVP-A, suggesting the production of a more robust gel-like structure. Furthermore, the G′ and G″ values of VVP-W were noticeably higher than those of VVP-A during the cooling process. As opposed to VVP-W and VVP-A, VVP-C showed noticeably lower G′ and G″ values during the cooling process. Also, the G′ and G″ values of VVP-C displayed negligible temperature variations, indicating a weak-gel behavior. The results show that solvent extraction significantly impacts the rheological properties of blackberry polysaccharides [21], which may be explained by its effect on elements like molecular weight and molecular chain conformation when combined with steady-state rheological analysis and oscillatory frequency sweep experiments.

### 3.7. Functional Properties of VVPs

#### 3.7.1. WHC and OHC

**Figure 6:**
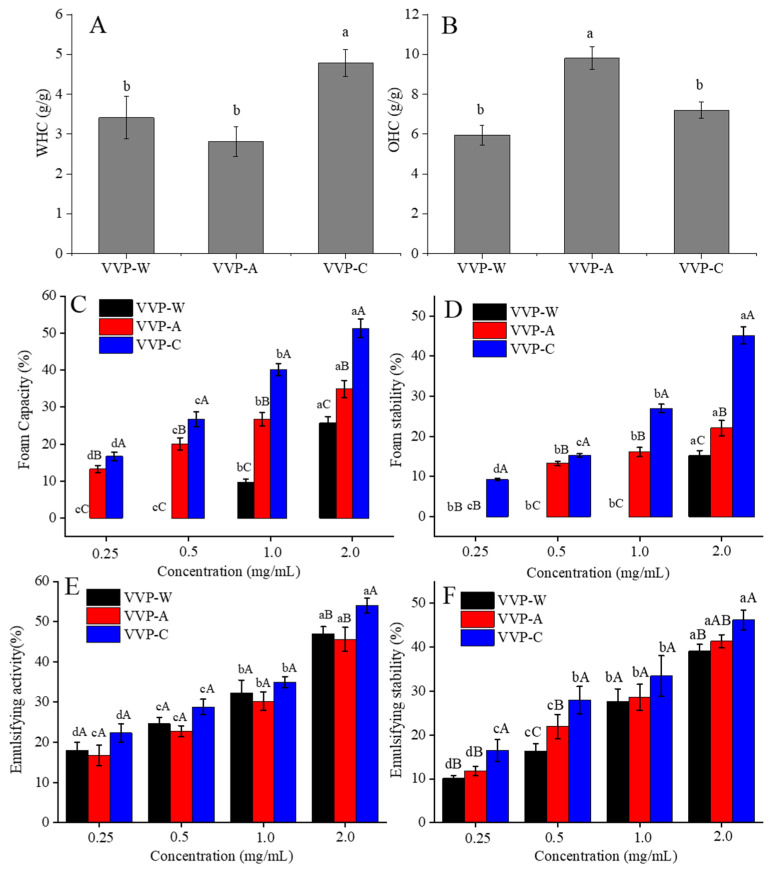
WHC (**A**), OHC (**B**), foaming capacity (**C**), foaming stability (**D**), emulsifying capacity (**E**), and emulsifying stability (**F**) of VVPs with different concentrations. Different lowercase letters in the superscripts indicate significant differences among same samples with different concentrations (*p* < 0.05); different capital letters in the superscripts indicate significant differences among different samples with the same concentrations (*p* < 0.05).

Figure 6A,B show the evaluation of the effects of various solvents on the WHC and OHC of the VPP samples. The WHC measures the ability of a sample to absorb and retain water [43]. According to our research, the extraction solvent selection has a significant impact on the WHC of VVPs (Figure 6A). The tested VVP samples, which ranged from 2.81 (g water/g) for VVP-A at ambient temperature to 4.78 (g water/g) for VVP-C, showed an outstanding WHC overall. This indicates that VVP has a substantially higher WHC than the other polysaccharides that are present in inulin (1.59 g water/g) and okara (0.27 g water/g) [44,45]. Due to their higher fermentability in the large intestine and ability to modify bulk volume, viscosity, texture, sensory assessment, and stool volume growth, high WHC fibers are essential to the food business [46]. A VVP extracted in a citric acid solution (VVP-C) has a high WHC. Hence, it could be used as a high WHC agent. Nevertheless, as Figure 2 illustrates, VVP-W samples had a low WHC comparable to VVP-A, even with their high molecular weight. Particle size, a crucial solubility component, may cause this discrepancy [47]. The hydration positions or active sites, pore size, conformational structure, and capillary properties of the molecule dictate the WHC [48].

As shown in Figure 6B, the extraction solvent selection affected the OHC of VVP. VVP-W demonstrated an oil-holding capability around six times greater than its weight. Significantly, compared to dietary fibers and polysaccharides, the OHC of the VVP samples extracted using the other two solvents was 1.21–1.65 times lower than that of VVP-A. In comparison, the OHC values of commercial pectin [49] and inulin [48], reported to be 2.1 g/g and 1.6 g/g, respectively, were surpassed by that of VVP-A, which absorbed about ten times its weight in oil. By reducing the absorption of dietary fats in the gut, food ingredients with high OHC, like VVP-C, can help to normalize aberrant blood lipid profiles and help with weight control [50]. They are also essential in food compositions as mouthfeel-enhancing agents and flavor retainers. The physical trapping of oil molecules is primarily responsible for the OHC [51]. Since surface properties are crucial for oil adsorption, the higher OHC of VVP-A may result from its more significant surface area. These results are consistent with the observations in the SEM micrographs discussed below.

#### 3.7.2. Foaming Properties

The dispersion of gas bubbles in water is called foam. Thus, surface-active compounds that are capable of adsorption at the water–air interface are necessary. Figure 6C,D display the foam capacity (FC) and foaming stability (FS) of the three VVPs (VVP-W, VVP-A, and VVP-C) at 2% concentration. VVP-C exhibited higher FC and FS than VVP-W, producing a more stable foam. VVP-C has the lowest molecular weight among the three VVPs, which helps to explain this. As a result, VVP-C has more solubility and hydrophilicity due to its smaller molecular sizes than VVP-W and VVP-A, which have larger molecular sizes. The air–water interface is where VVP-C is more likely to adsorb, which increases the foaming capacity and foam stability. Furthermore, more soluble protein molecules in VVP-C could diffuse to the air–water interface, lowering the surface tension and promoting foam generation [51]. As a result, these variables cause VVP-C to have a higher foaming capacity and foam stability than VVP-A and VVP-W. Sarker et al. (1998) investigated the impact of adding 0.2–0.3 mg/mL of arabinoxylan dispersions to bovine serum albumin. They discovered that this increased the stability of the protein foam by creating wheat arabinoxylan-protein cross-links [52].

#### 3.7.3. Emulsion Properties

Two characteristics can be used to describe the emulsifying properties of an emulsion, which is a mixture of two liquids that are usually immiscible: EC and ES. These properties are crucial to polysaccharide function. This study used polysaccharide–water solutions of 0.25%–2% (*w*/*v*) to produce emulsions. Figure 6E,F present the results. The EC and ES of the three VVP samples displayed a dose-dependent pattern. VVPs had a poor emulsifying activity and stability at low concentrations, but as the polysaccharide concentration increased, their emulsifying properties considerably improved. VVP-C showed the highest emulsifying activity at 54.2% at a concentration of 2%, whereas VVP-A showed the lowest emulsifying activity at 45.6%. There was a slight difference in the emulsifying activity of three VVPs at a concentration of 2%. Moreover, the emulsifying stability of VVPs varied within the range of 0.25%–2.0%, with VVP-C exhibiting the highest emulsifying stability, followed by VVP-W and VVP-A. When comparing the overall emulsifying capacity and stability of three VVPs, it was found that VVP-C outperformed VVP-W and VVP-A as emulsifiers in food items. This discrepancy might be ascribed to the VVP extraction solvent used and other components, like ferulic acid, proteins, and minerals, which could contaminate the extracted fraction. The functional characteristics of polysaccharides are primarily determined by their structure. Because proteins and ferulic acid are hydrophobic, they decrease the interfacial tension between water and oil droplets, increasing the emulsion capacity [53]. Additionally, the presence of galactose in the side chains positively impacts the emulsion qualities, which connect the leading chains to ferulic acid and proteins [54].

### 3.8. α-Amylase and α-Glucosidase Inhibitory Capacities of VVPs

**Figure 7:**
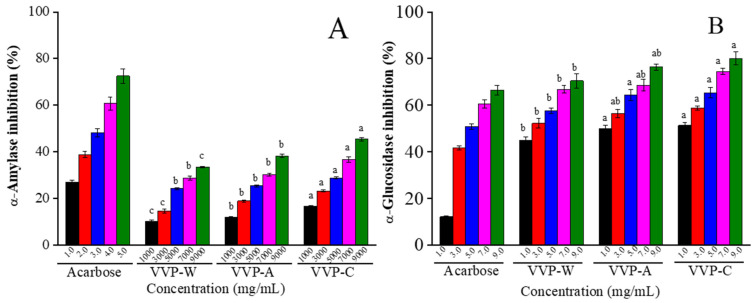
Inhibitory effect of VVPs on α-amylase (**A**) and α-glucosidase activity (**B**). Acarbose was used as the positive control. Different lowercase letters in the superscripts indicate significant differences among the different samples with the same concentrations (*p* < 0.05).

To regulate glucose absorption, type 2 diabetes is frequently treated with the inhibition of α-amylase and α-glucosidase activity [55]. Figure 7A,B illustrate how VVPs affect the activity of α-amylase and α-glucosidase. These tests offer a quick and easy approach to assessing the hypoglycemic properties of bioactive compounds in vitro [27]. Similar dose-dependent inhibitory effects were shown by all three VVPs on the activities of α-amylase and α-glucosidase activities. The inhibition was demonstrated by VVP-C, which was extracted using a citric acid solution. Nevertheless, compared to acarbose, VVP-C had less of an inhibitory effect. VVP-C reduced α-glucosidase activity by 80.05% and α-amylase activity by 45.44% at a concentration of 9.0 mg/mL. This indicates that VVP-C inhibits α-glucosidase more potently than α-amylase. One possible explanation for the variation in inhibitory strength could be the distinct modes of action exhibited by α-glucosidase and α-amylase. As such, it is preferable to have a mild inhibition of α-amylase and a higher inhibition of α-glucosidase [56]. Similarly, at the higher concentrations necessary for α-amylase inhibition, the polysaccharides extracted from *Dioscorea hemsleyi* inhibited both α-glucosidase (half inhibitory concentration, IC_50_ of 27.41–274.36 μg/mL) and α-amylase (IC_50_ of 3.66–47.57 μg/mL) [57]. However, in contrast to α-amylase, polysaccharides from *Sargassum pallidum* had less inhibitory effects on α-glucosidase [18]. The molecular weight and chemical structures of VVPs, sugar and protein content, monosaccharide composition, and other variables can affect the inhibitory actions of α-glycosidase and α-amylase [58]. In conclusion, compared to other solvents, citric acid extraction is more successful at removing VVPs from VV with notable antioxidant and hypoglycemic properties.

## 4. Conclusions

This study used a hot water, a NaOH solution, and a citric acid solution to extract VVPs. Among them, VVP-W showed the highest molecular weight and best colloidal properties, suitable for food thickening. VVP-A exhibited the most promising oil-holding capacity, conducive to the application of powdered oil. VVP-C presented the most desired foaming, emulsion, and hypoglycemic activities, available as emulsifiers and nutritive food additives. This study could provide guidance for selecting appropriate extraction methods using sustainable unconventional processes to improve the extraction yield and purity of polysaccharides from *Volvariella volvacea*, minimize energy consumption, and produce functional polysaccharides with novel bioactive properties as food additives.

## Figures and Tables

**Table 1:** Extraction yields and chemical compositions of VVP-W, VVP-A, and VVP-C.

	VVP-W	VVP-A	VVP-C
Extraction yield (%)	5.15 ± 0.33 ^b^	7.68 ± 0.51 ^a^	6.31 ± 0.41 ^a,b^
Total sugar content (%)	81.65 ± 2.10 ^c^	85.70 ± 1.75 ^b^	87.69 ± 1.56 ^a^
Total phenolic content (mg GAE/g)	26.04 ± 2.90 ^a^	18.41 ± 3.13 ^b^	23.05 ± 1.95 ^a^
Total protein content (%)	1.98 ± 0.17 ^b^	1.41 ± 0.11 ^b^	2.77 ± 0.25 ^a^
Molecular weight (*M_w_*, kDa)	23.3	20.7	19.3
Monosaccharide composition (molar ratio, %)			
Fucose	4.00	2.90	4.20
Galactose	18.40	16.70	21.10
Glucose	77.60	80.40	74.70

Note: Different lowercase letters in the same row were used to represent statistical significance among different samples (*p* < 0.05).

## Data Availability

The datasets generated for this study are available upon request from the corresponding author.
